# Environmentally specific servant leadership and voluntary pro-environmental behavior in the context of green operations: A serial mediation path

**DOI:** 10.3389/fpsyg.2022.1059523

**Published:** 2022-11-07

**Authors:** Hina Zafar, Feng Tian, Jo Ann Ho, Gaoqi Zhang

**Affiliations:** ^1^Newcastle Business School, The University of Newcastle, Callaghan, NSW, Australia; ^2^School of Business and Economics, Universiti Putra Malaysia, Selangor Darul Ehsan, Malaysia; ^3^Business School, Nanjing Xiaozhuang University, Nanjing, China

**Keywords:** voluntary pro-environmental behavior, environmentally specific servant leadership, organizational identity, green operations, psychological empowerment

## Abstract

Green operations of organizations and enhancement of corporate social responsibility hinges upon leaders. This study investigated the influential role performed by environmentally specific servant leadership in provoking voluntary pro-environmental behavior of employees. The findings illuminate a serial chain mediation model that originates as a result of environmentally specific servant leadership and leads toward psychological empowerment, and organizational identity, ultimately leading toward voluntary pro-environmental behavior. Data from the textile sector of Pakistan uncovered that environmentally specific servant leadership prompted the voluntary pro-environmental behavior of employees. Moreover, environmentally specific servant leadership was significantly linked with voluntary pro-environmental behavior through psychological empowerment. The study supports the serial mediation of psychological empowerment and organizational identity in stirring voluntary pro-environmental behavior. An organizational psychological mechanism has been unraveled that can help organizations achieve a high level of sustainability and can serve as a catalyst for organizational green operations.

## Introduction

Organizations have started realizing the connection between corporate social responsibility and organizational effectiveness (Afsar et al., 2018). Employees are the main actors in adopting and implementing green practices in organizations (Banwo and Du, 2019). There is a need to engage in pro-environmental behaviors to foster green operations of the organization and take measures to meaningfully employ human resources for the health and recovery of Earth Planet (Khan, 2018). Morren and Grinstein (2016) stated that people of developed countries are more involved in initiating voluntary green behaviors as compared to developing countries. Lülfs and Hahn (2013) acknowledge that employees of an organization play a substantial role in the greening of the organization through indulging in pro-environmental behaviors. Organizations around the globe have implemented different pro-environmental initiatives in the form of standardized environmental policies and procedures, compliance with environmental legislation, saving energy, reduction in water consumption, and recycling due to an alarming increase in environmental pollution (Zibarras and Coan, 2015). Voluntary pro-environmental behavior (VPEB) involves the voluntary participation of employees in the protection of the natural environment beyond their job expectations (Lamm et al., 2013). These voluntary behaviors are considered significant contributors to enhancing the environmental performance of organizations (Blok et al., 2015; Lamm et al., 2015; Robertson and Carleton, 2017; Silva, 2017; Xiao et al., 2020). These behaviors are not only important for environmental performance but also the performance of the organizations and their employees (Rivera and de Leon, 2004; Norton et al., 2015; Tuan, 2019a) because voluntary green behavior is consistent with the environmentally and socially responsible values, goals and beliefs of the organization and is significant for the success of the organization (Chou, 2014; Silva, 2017; Tian and Robertson, 2017; Mouro and Duarte, 2021). Eco-initiatives by employees like reduced water consumption, saving energy, and recycling behaviors can help in dealing with environmental issues (Boiral and Paillé, 2011). However, despite the growing interest of researchers in pro-environmental behaviors (Paillé et al., 2014; Norton et al., 2015), there is still a need to explore a combination of organizational, contextual, and individual-level factors that can enhance the pro-environmental behavior of employees (Lamm et al., 2015; Dumont et al., 2017; Saeed et al., 2019).

Leaders have the ability to significantly influence the voluntary green participation of employees (Robertson and Carleton, 2018). Servant leaders work altruistically for the benefit of their community and their followers, putting aside their own interests (Avolio et al., 2009; Rashid and Ilkhanizadeh, 2022). Similarly, environmentally specific servant leadership (ESSL) prioritizes the protection of the natural environment and makes the employees behave altruistically in terms of sustainability (Tuan, 2019a). Servant leadership has been reported to significantly shape the citizenship behavior of employees (Newman et al., 2017). Voluntary green behavior is a citizenship behavior as well (Jahanshahi et al., 2021). Therefore, the present research takes into consideration ESSL which can affect the voluntary green behavior of employees because of the altruistic nature of this leadership style. Although some studies have investigated the impact of ESSL on VPEB (Afsar et al., 2018; Luu, 2019), such research within the manufacturing sector of Pakistan has not been done yet. ESSL role models the corporate social responsibility values and employees can see the translation of these values into green actions (Afsar et al., 2018). Thus, employees view their organization as environmentally responsible and desire to attach to the organization (Afsar et al., 2018).

Spreitzer (1995) identified four dimensions of empowerment, named self-determination, impact, competence, and meaning. Meaning defines the fit between a person’s work goals and values or beliefs. Competence defines the beliefs of employees that they can skillfully perform their tasks. Self-determination is involved in sense of autonomy of employees and control over their work. The impact defines the extent that individuals believe that their every action is making a difference. Psychologically empowered employees view work as a valuable source and are more likely to involve in reciprocal behavior in the form of lower turnover intention and loyalty with the organization (Blau, 1964). Members of the organization realize that work empowerment is difficult to find, so the search for finding a better arrangement is lowered (Seibert et al., 2011). Psychological empowerment allows employees of the organization to believe that they have complete control over their work (Maynard et al., 2012).

A recent study by Zafar et al. (2022b) highlighted the mediation of organizational identity between ESSL and VPEB. However, the intervening mechanism of psychological empowerment between ESSL and organizational identity is missing. So far, green organizational climate (Tuan, 2019a; Zafar et al., 2022a), green crafting (Tuan, 2019a), and organizational identity (Zafar et al., 2022b) are among the few predictors that have been reported as direct predictors of ESSL. Two notable studies by Newman et al. (2017) and Ying et al. (2020) investigated the impact of servant leadership on psychological empowerment. However, the impact of ESSL on psychological empowerment and the outcome of organizational identity and VPEB as a result of psychological empowerment is still missing in sustainability literature. The impact of servant leadership on psychological empowerment in a sustainability context should be explored for enhancing the green behavior of employees (Ying et al., 2020), which can ultimately serve to enhance the green operations of the organization. The link between psychological empowerment and organizational identity has been identified by previous researchers (Avan et al., 2019; Bose et al., 2021). However, ESSL as a predictor of psychological empowerment and organizational identity as an intervening variable between psychological empowerment and VPEB are notable contributions of the present research. So far, the researchers have identified ESSL as a predictor of very few focal variables such as green performance (Tuan, 2019b) and green creativity (Tuan, 2019a). Although recent research has begun to examine the effect of ESSL on VPEB (Luu, 2019; Tuan, 2019a; Zafar et al., 2022b). However, it has not looked at the intervening mechanism that links ESSL to VPEB. The present research identifies based on social identity theory that ESSL provides employees with the opportunity to inspire them for participation in green tasks (Tuan, 2019a). Thus, they feel more competent, confident and find their work meaningful as a result of the leader’s support (Newman et al., 2017). Hence, the more empowered employees are more satisfied with their work and are more likely to identify with their organization. Feelings of competence and self-determination motivate them to identify with the organization. Being a part of a green organization enhances pride among employees (John et al., 2019; Ng et al., 2019; Zafar et al., 2022a). Consequently, they feel more encouraged to carry out voluntary initiatives that are of great significance to the organization (Jones et al., 2014).

The textile industry is among the most pollution-causing industries in the world and demand for textile products is surging day by day (Hayat et al., 2020). The industry is responsible for 10% global greenhouse gas emissions (Razzaq et al., 2018). It has been anticipated by 2050, this sector will utilize up to 25% of the global carbon budget (Chen et al., 2021). To deal with the destructive effects of the textile sector, there is a pressing need to transform this sector into a sustainable sector (Zafar et al., 2022b). The textile sector is the most significant sector of Pakistan and contributes 60% to its exports (Zafar et al., 2022a). At the global level, Pakistan stands 4th among the largest cotton-producing countries in the world (Hayat et al., 2020). The majority of the studies on ESSL have been conducted in the hospitality industry (Luu, 2019; Tuan, 2019a; Zheng et al., 2021) and neglected its role in the textile sector. It is crucial to investigate the impact of ESSL in the textile sector for finding ways of encouraging VPEB among employees (Zafar et al., 2022b). Textile is considered the secondary industry and is responsible for huge carbon dioxide emissions (Nature Climate Change, 2018; Zafar et al., 2022b). The focus on green development is higher in western countries as compared to eastern countries (Ding et al., 2022). As Pakistan is an eastern country and has a significant textile sector, so it is critical to investigate on how this sector can be transformed into a sustainable sector (Zafar et al., 2022b). For the development of successful green operations of the organization, it is crucial to focus on the green altruistic behavior of employees (Yong et al., 2019). The present research argues that the role of employees in accelerating the green performance of the organization needs to be investigated (Safavi and Bouzari, 2019). Employees can play a significant role in enhancing the green operations of the organization and researchers have not elaborated well that how ESSL can accelerate green organizational performance (Luu, 2020). Thus, the present study intends to make a significant contribution to the sustainability literature by elaborating on how ESSL can help in accelerating the green operations of the organization by provoking voluntary green initiatives of employees.

The framework of the present study draws on the social exchange (Blau, 1964) and social identity (Tajfel, 1978) theories for the development of the theoretical foundation of the present study. Based on the social exchange theory, ESSL provides employees with sufficient resources to take part in green tasks that make the employees feel obliged to reciprocate with enhanced efforts (Zafar et al., 2022b). Servant leaders enhance the self-efficacy of employees and equip them with autonomy over their activities, enhancing their confidence. In line with social identity theory, enhanced self-esteem stimulates the voluntary engagement of employees (Mael and Ashforth, 1992). An employee drives his identity from his workgroup. Being a part of a green organization boosts the pride of an employee for demonstrating care for the community (Jones, 2010). Resultantly, the employee responds with enhanced dedication and effort.

Overall, the study enriches the sustainability literature by examining the link between ESSL and VPEB within the context of Pakistan’s textile sector. The mediation of psychological empowerment between ESSL and VPEB, between ESSL and organizational identity, is another contribution of the present study. The serial mediation chain model in the form of psychological empowerment and organizational identity between ESSL and VEPB is also notable.

## Hypotheses development

### ESSL and VPEB

ESSL prioritizes environmental concerns and gains over the financial benefits of the organization and mainly focuses on green values cultivation among employees (Afsar et al., 2018). Environmentally specific servant leaders inspire employees and provide them with significant knowledge and skills to take part in green endeavors (Afsar et al., 2018). These leaders encourage and appreciate the sustainable efforts of their employees and engage them with new green projects for enhancing their green competency (Luu, 2019). Based on social exchange theory (Blau, 1964), leaders prioritize the sustainable interests of employees and provide support and resources to them to take part in green tasks. Resultantly, employees feel obliged and reciprocate with enhanced efforts. When an organizational leader provides employees with sufficient resources to engage in green tasks (Tuan, 2019a), a high-quality relation is established between the employee and the leader that makes the employee feel obliged to repay the favors that have been positively conferred on him (Cole et al., 2002; Thompson et al., 2021; Zafar et al., 2022b). Therefore, it can be anticipated that:

*H1*: ESSL positively affects the VPEB of employees.

### ESSL, psychological empowerment, and VPEB

Servant leadership has been reported to have a significant impact on psychological empowerment because they treat their employees with emotional support and respect and make them feel enhanced meaning in their work (Newman et al., 2017). ESSL enhances the confidence of employees by providing them with essential resources, skills, and knowledge to carry out sustainable tasks (Tuan, 2019b). By providing the opportunity to followers for learning new skills and training them to engage with proactive tasks, ESSL fosters competency among employees (Newman et al., 2017). Thus, ESSL makes the employees psychologically empowered. In terms of social exchange theory (Blau, 1964), the more empowered employees are more action-oriented toward their work and perform beyond their job description, thus carrying out voluntary green initiatives actively. Psychologically empowered employees are more motivated, more focused on work, and highly resilient (Spreitzer, 1995). Servant leaders enhance the self-efficacy of employees and equip them with the feeling that they have complete freedom to execute their tasks (Ying et al., 2020). Thus, they go beyond their job expectations to perform tasks that are of significance to the organization. The present study proposes that environmentally specific servant leaders empower the employees with the feeling that they have complete freedom to perform green activities and provide them with green knowledge and skills (Luu, 2020). Thus, they feel more motivated to engage in green activities for serving organizational green tasks (Tuan, 2019a; Luu, 2020). Hence, it can be said that:

*H2*: Psychological empowerment mediates the relationship between ESSL and VPEB of employees.

### ESSL, organizational identity, and VPEB

Environmentally specific servant leaders support the green actions of employees and encourage their participation in green projects (Afsar et al., 2018). Leaders create a safe environment within the organization and encourage employees as a mentor to work on challenging projects and allow organizations to cultivate the “interest” of employees and lead to the development of a shared mental model (Tuan, 2019b). By providing support and resources to employees and communicating the vision of the organization, leaders promote a shared perspective among employees that the organization values the environmental contribution of its workers (Priyankara et al., 2018; Tuan, 2019b). In this way, a high-quality relationship is established between employees and leaders. Being a part of a sustainable organization enhances pride among employees because it ultimately enhances employees’ health and well-being (Zafar et al., 2022a). Environmental leaders have been reported to motivate employees to identify with the organization (Al-Ghazali et al., 2022; Zafar et al., 2022b). Consequently, employees feel more motivated to identify with the organization and proactively engage with VPEB. In line with social identity theory (Tajfel, 1978), when a leader develops a strong bond with employees, he makes the employees feel like a partner in the organization. Thus, a leader enhances employees’ sense of belongingness and identity (Teng et al., 2020). This identity motivates the employees to take part in behaviors that are beneficial to the organization (Tajfel, 1978; Teng et al., 2020). Thus, it can be proposed that:

*H3*: Organizational identity mediates the relationship between ESSL and VPEB of employees.

### ESSL, psychological empowerment, and organizational identity

ESSL provides significant support to employees for green activities and provides them with enhanced knowledge and skills to lighten their minds with the importance of sustainability (Tuan, 2019b). Resultantly, employees would be highly psychologically empowered, more motivated, responsible, and connected to work (Zhang and Bartol, 2010). Concerning social identity theory (Mael and Ashforth, 1992), more psychologically empowered employees as a result of ESSL would be more motivated to identify with the organization. Psychological empowerment serves as an intrinsic motivation factor for employees and enhances their confidence that they are performing meaningful work in the organization (Iqbal et al., 2020; Ying et al., 2020; Zafar et al., 2022b). Such employees would be clearer in terms of organizational objectives and realize that greening is a vital part of the organization and their green efforts would be appreciated by the organization. As sustainable organizations have a good reputation in the eyes of the community, employees feel honored to be a part of such an organization and feel pride in their organizational membership, hence their organizational identity is enhanced (Ng et al., 2019; Kim et al., 2020; Zafar et al., 2022a). Thus, it can be argued that:

*H4*: Psychological empowerment mediates the positive relationship between ESSL and organizational identity.

### ESSL, psychological empowerment, organizational identity, and VPEB

As discussed above, ESSL equips the employees with the feeling that they are doing meaningful work and their work will be appreciated by the organization (Tuan, 2019b). Hence, their psychological empowerment is enhanced. It has been reported that servant leaders nurture the psychological empowerment of employees by offering them autonomy in their work domains, treating them equally in a transparent way (Ying et al., 2020), and enhancing their green awareness (Luu, 2019). These leaders focus on employees’ development instead of treating them as a source of advantage for the organization (Ying et al., 2020). Environmentally specific servant leaders equip the employees with the necessary knowledge and resources to enhance their awareness (Tuan, 2019a, 2019b; Zafar et al., 2022b). Higher autonomy, competence, and connectedness with the organization expand the approach of employees and they feel more connected to the organization (Iqbal et al., 2020), thus strengthening their identity. High psychological empowerment would make the employees confident to work on challenging tasks to achieve the objectives of the organization (Spreitzer, 1995; Avolio et al., 2004). Employees would feel the green objectives of the organization as attractive because sustainability is ultimately related to the well-being of employees (Kim et al., 2020; Zafar et al., 2022a). Employees are always willing to put forth their efforts on the organization’s behalf and would accomplish the goals that are valued by the organization (Daily et al., 2009). Thus, the following can be proposed:

*H5*: Psychological empowerment and organizational identity sequentially mediate the positive relationship between ESSL and VPEB.

**Figure 1:**
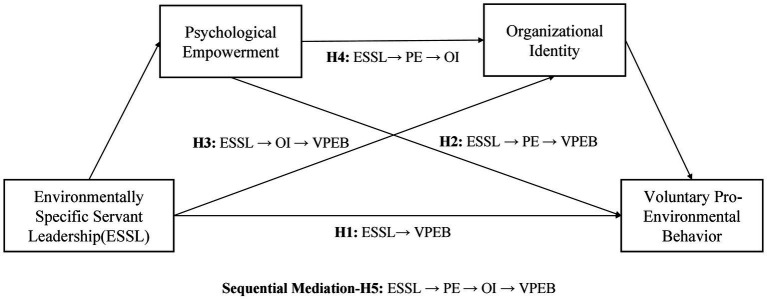
Research framework.

Based on the above discussion, the following research framework has been developed (Figure 1).

## Methodology

Voluntary Pro-environmental behavior was assessed utilizing a 12-item scale of Lamm et al. (2013). Organizational identity was evaluated using a scale derived from Mael and Ashforth (1992). Psychological empowerment was examined using a 12-item scale of (Spreitzer, 1995). Environmentally specific servant leadership was evaluated using seven items adapted from Liden et al. (2014) and Liden et al. (2008).

### Participants and procedure

The list of textile firms was obtained from the Security and Exchange Commission website of Pakistan. Our respondents were subordinates and their direct supervisors. The present research selected respondents for data collection who fit in the criteria for the selection of employees and managers, following purposive sampling. Those employees whose profile was not according to the set criteria for the present research were rejected and they were not part of the research. Data were collected from only those employees who had at least 2-year experience of working under the supervision of the manager because the more time they would have spent with the organization, the more they would have an understanding of policies, practices, and the work environment of the organization. Further, the more time the employees spent with managers, the more accurately the managers would be able to rate their behavior. This is in line with studies conducted by Ying et al. (2020), Saeed et al. (2019), and Afsar et al. (2018). Data were collected from medium- and large-sized organizations because these organizations have enough resources to adopt green practices (Samad and Ahmed, 2015). Small organizations were not included because they would not have sufficient resources to adopt green practices (Samad and Ahmed, 2015). Meetings were arranged with the managers to make them aware of the study and employees were ensured of the confidentiality of their responses.

G power statistical analysis was conducted that suggested a sample size of 217. The data were collected utilizing purposive sampling and 384 has been reported as enough sample size for an infinite population (Krejcie and Morgan, 1970). Previous studies have reported a response rate between 40% and 70% in the manufacturing sector (Afsar et al., 2018; Das et al., 2019). Keeping in mind this response rate, 800 questionnaires were distributed among textile employees of 32 companies located in the Punjab province of Pakistan. One hundred and twenty-nine managers were invited to respond on the VPEB of employees. Managers provided separate responses for the behavior of each employee. Employees responded to all the criterion variables. Out of 800 questionnaires, 570 were returned, out of which 434 were usable responses. Socially desirable responses by employees can lead to a fictitious relationship between variables and affect the behavior and attitude related to issues of the environment (King and Bruner, 2000; Larson, 2019). For avoiding common source bias, direct supervisors of employees were asked to rate the VPEB. Employees were requested to write their names on the questionnaires so that the responses could be matched with their supervisory ratings. This type of data collection is in line with the study of Afsar et al. (2017), Afsar et al. (2020), and Saeed et al. (2019).

About 82% of the data were gathered from large, while 18% was from medium textile firms. The organizational size was measured based on the number of employees. Organizations having employees between 50 and 250 were considered medium while having more than 250 were considered large organizations. All the managers were having a postgraduate level of education. 62% of the employees were graduates or above level of education.

The firm size (control variable), which has been previously identified to influence voluntary pro-environmental behavior (Tuan, 2019a), was determined based on the number of employees in the organization.

### Data analysis

Partial least square structural equation modeling was utilized and Smart PLS software was used for analyzing the data for the present study. PLS-SEM was appropriate to use in our study because the goal of our study is to explore and examine the proposed research model (Sarstedt and Cheah, 2019). In addition, the prediction-oriented objective of the current research can be achieved by the causal prediction approach of PLS-SEM (Chin et al., 2020; Hwang et al., 2020). Higher-order constructs can be assessed well with PLS-SEM (Hair et al., 2019; Sarstedt et al., 2019) and mediation effects can be better analyzed with PLS-SEM (Nitzl et al., 2016). The following sections represent the results for the reflective and structural measurement model.

### Results of the reflective measurement model

The data confirmed the convergent validity and internal consistency including the composite reliability, Cronbach alpha in line with recommendations by Hair et al. (2017). Results can be seen in Table 1. Composite reliability was not less than 0.70, average variance extracted was equal to and greater than 0.50. Results can be observed in Table 1.

**Table 1:** Convergent validity and internal consistency.

Constructs	Items	Loading	CA	Rho_A	CR	AVE
Environmentally specific servant	ESSL1	0.86	0.86	0.87	0.89	0.55
Leadership	ESSL2	0.74				
	ESSL3	0.71				
	ESSL4	0.77				
	ESSL5	0.56				
	ESSL6	0.77				
	ESSL7	0.73				
Organizational identity	OI1	0.72	0.80	0.82	0.86	0.50
	OI2	0.71				
	OI3	0.59				
	OI4	0.80				
	OI5	0.74				
	OI6	0.67				
Meaning	PE1	0.88	0.85	0.85	0.91	0.77
	PE2	0.91				
	PE3	0.84				
Self-determination	PE4	0.90	0.90	0.90	0.94	0.83
	PE5	0.91				
	PE6	0.91				
Impact	PE7	0.82	0.78	0.78	0.87	0.70
	PE8	0.81				
	PE9	0.87				
Competence	PE10	0.83	0.71	0.72	0.84	0.63
	PE11	0.80				
	PE12	0.76				
Voluntary pro-environmental behavior	VPEB1	0.78	0.92	0.95	0.93	0.54
	VPEB2	0.80				
	VPEB3	0.74				
	VPEB4	0.81				
	VPEB5	0.75				
	VPEB6	0.82				
	VPEB7	0.76				
	VPEB8	0.65				
	VPEB9	0.81				
	VPEB10	0.66				
	VPEB11	0.58				
	VPEB12	0.58				

AVE, Average variance extracted; CA, Cronbach alpha; CR, Composite reliability.

The results further confirmed the Heterotrait-Monotrait ratio (HTMT) values for the discriminant validity (Hair et al., 2017). None of the values were greater than 0.90, thus confirming discriminant validity (see Table 2A). The Fornell–Larcker criterion further confirmed the discriminant validity of the present study. It can be observed in Table 2B that the square root of the average variance extracted of each variable is higher as compared to its squared correlations with other variables of the model (Fornell and Larcker, 1981).

**Table 2A:** Assessment of discriminant validity.

Constructs	1	2	3	4
1. Environmentally specific servant leadership				
2. Organizational identity	0.55			
3. Psychological empowerment	0.54	0.48		
4. Voluntary pro-environmental behavior	0.54	0.51	0.40	

**Table 2B:** Fornell–Larcker criterion.

Constructs	1	2	3	4
1. Environmentally specific servant leadership	**0.74**			
2. Organizational identity	0.49	**0.71**		
3. Psychological empowerment	0.48	0.43	**0.86**	
4. Voluntary pro-environmental behavior	0.52	0.49	0.39	**0.73**

Bold values represent square root of AVE.

### Result of the higher-order constructs

Psychological empowerment was treated as a formative construct. Variance inflation factor values for the four dimensions of psychological empowerment were not greater than 3.3 and all the dimensions were statistically significant (Table 3).

**Table 3:** Measurement properties of formative construct.

Higher-order construct	Dimensions of lower order construct	Outer weights	Outer loadings	Value of *p*	VIF
PE	Meaning	0.28	0.85	0.00	2.30
	Self-determination	0.29	0.88	0.00	2.70
	Impact	0.30	0.90	0.00	2.92
	Competence	0.28	0.81	0.00	1.88

PE, Psychological empowerment; VIF, Variance inflation factor.

### Results of the structural model

**Figure 2:**
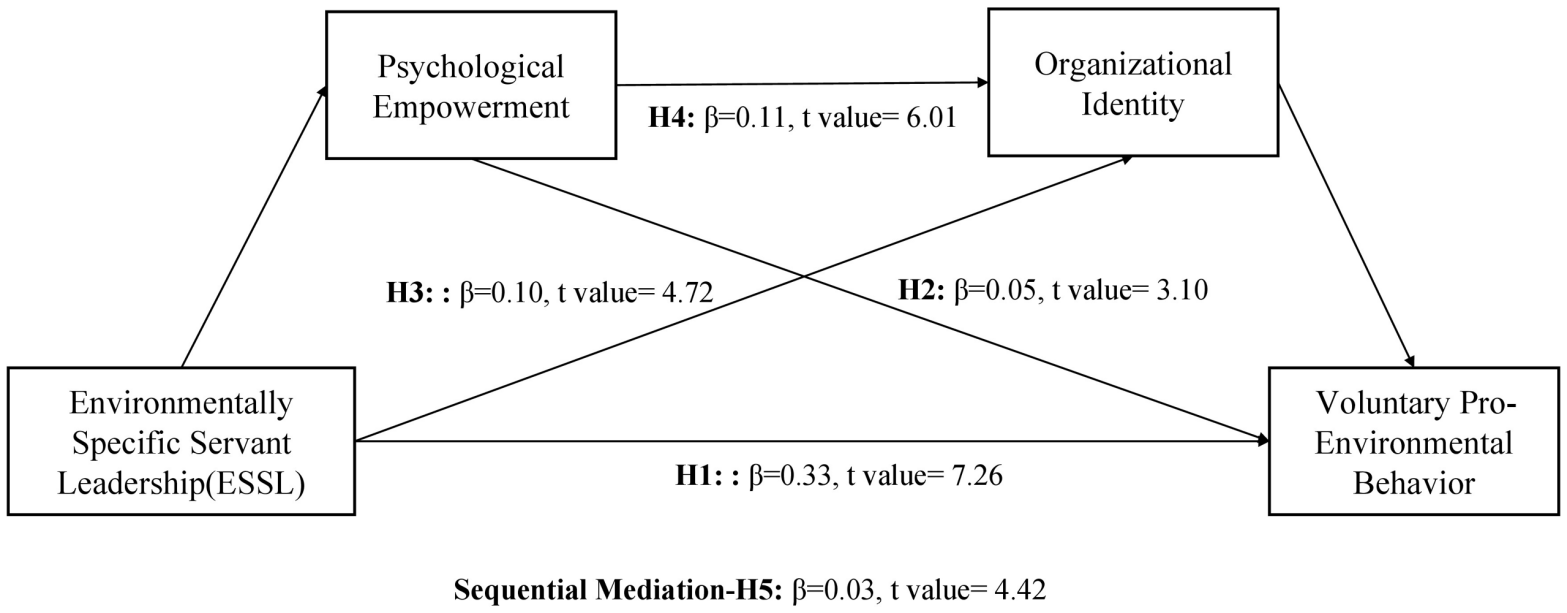
Structural Model.

Following Figure 2 represents the structural model of the present study.

The coefficient of determination (R^2^) was examined for evaluating the explanatory power of constructs (Hair et al., 2017). The R^2^ value of 0.22 for psychological empowerment demonstrated that ESSL accounted for 22% of the variance. 28% of the variance in organizational identity was expounded by psychological empowerment (R^2^ = 0.28) and organizational identity explained 35% of the variance in VPEB. Additionally, a 1% change was observed in the R^2^ of VPEB after eliminating the control variable of firm size, indicating no issue of common method bias in the present study (Hair et al., 2017).

Table 4 provides the summary of hypotheses results. ESSL was significantly linked with voluntary pro-environmental behavior (H1; β = 0.33, *p* ≤ 0.05), thereby providing support for hypothesis H1. Furthermore, the results of Table 4 show that psychological empowerment mediated the influence of ESSL on voluntary pro-environmental behavior (H2; β = 0.05, *p* ≤ 0.05). ESSL indirectly influenced voluntary pro-environmental behavior through organizational identity (H3; β = 0.10, *p* ≤ 0.05). Psychological empowerment was found to positively mediate the relationship between ESSL and organizational identity (H4; β = 0.11, *p* ≤ 0.05). The results further confirmed the serial mediation of psychological empowerment and organizational identity between ESSL and VPEB (H5; β = 0.03, *p* ≤ 0.05). Furthermore, firm size non-significantly affected VPEB (β = −0.04, *p* = 0.14).

**Table 4:** Hypotheses results.

Relationships	β	SE	*t*-value	Value of *p*	CIs (5.0–95.0%)	Results
**Mediation analysis**						
ESSL ➔ PE ➔ VPEB	0.05	0.01	3.10	0.00	(0.02; 0.08)	Accepted
ESSL ➔ OI ➔ VPEB	0.10	0.02	4.72	0.00	(0.06; 0.13)	Accepted
ESSL ➔ PE ➔ OI	0.11	0.02	6.01	0.00	(0.08; 0.15)	Accepted
**Direct Effect**						
ESSL ➔ VPEB	0.33	0.04	7.26	0.00	(0.25; 0.40)	Accepted
**Serial mediation analysis**						
ESSL ➔ PE ➔ OI ➔ VPEB	0.03	0.02	4.42	0.00	(0.02; 0.04)	Accepted
**Control variable**						
Firm size	−0.04	0.03	1.07	0.14	(−0.10; 0.02)	Non-Sig

ESSL, Environmentally specific servant leadership; VPEB, Voluntary pro-environmental behavior; PE, Psychological Empowerment; OI, Organizational identity; CIs, Confidence intervals; SE, Standard error; *p*-value < 0.05; Non-Sig, Non-significant.

## Discussion

Overall, the study provides support to serial mediation of psychological empowerment and organizational identity between ESSL and VPEB. The Association of ESSL with VPEB through the mediation of psychological empowerment and mediation of organizational identity has also been confirmed by the present study. The direct impact of ESSL on VPEB has also been confirmed. The following sections present the theoretical and practical implications of the current research.

### Theoretical implications

By investigating the impact of ESSL on VPEB, the present study responded to calls for research by Ying et al. (2020) and Robertson and Barling (2017) who invited future scholars to explore the impact of ESSL on voluntary workplace green behavior of employees. The present study indicates that ESSL can provoke the motivation of employees to indulge in green initiatives (Robertson and Barling, 2017; Afsar et al., 2018). Studies by Luu (2020) and Tuan (2019a) acknowledge that ESSL can play a significant role in enhancing the green performance of the organization. ESSL can provoke the green behavior of employees which ultimately fosters the development of green operations of the organization (Luu, 2020).

The current results highlight the important impact of psychological empowerment and organizational identity on the voluntary green behavior of employees. The findings of our study recommend that more psychologically empowered employees as a result of ESSL enhance their self-esteem which motivates them to identify with the organization and enhance their voluntary green initiatives. Although researchers are giving attention to antecedents of voluntary green behavior, the research on multilevel antecedents of voluntary pro-environmental behavior is still in its infancy (Afsar et al., 2018; Aboramadan et al., 2021). Therefore, current research augments sustainability literature by exploring the connection between organizational (ESSL) and individual (psychological empowerment, organizational identity, voluntary pro-environmental behavior) variables. Furthermore, our study confirmed that psychological empowerment comprising competence, self-determination, and perception of meaningful work of employees significantly created the employees’ organizational identity. Thus, the present study enriches the studies by Newman et al. (2017), Ying et al. (2020), Zafar et al. (2022b), and Tuan (2019a) by introducing psychological empowerment and organizational identity as intervening variables between ESSL and VPEB. Hence, our study provides support to previous studies (Kahaleh and Gaither, 2007; Bose et al., 2021) which posit a profound relationship of psychological empowerment with the employee’s identity. The study confirms that as a result of ESSL, individual personality traits in the form of psychological empowerment enhance the identity of employees. This identity, in turn, motivates the employees to carry out voluntary green initiatives. The current study brings into light the significant impact of psychological empowerment and organizational identity as important serial mediators between ESSL and VPEB based on social identity and social exchange theories.

Lastly, this research in Pakistan enriches the literature on ESSL within the context of Asia. Pakistan stands 12th in the World in the list of countries (Ullah, 2017) that have been harshly influenced by environmental degradation (Khan et al., 2016; Ullah, 2017). In addition, the clothing industry stands second in terms of contaminator of the natural environment and is accountable for 10% of carbon emissions around the world (Razzaq et al., 2018). The present study responds to calls by Priyankara et al. (2018) and Tuan (2019a) by examining voluntary pro-environmental behavior within the context of Pakistan’s manufacturing sector. The present study unraveled the psychological mechanism for enhancing green functioning of the organization by fostering employees’ green behavior.

### Practical implications

The current research model proposes a pathway toward sustainability for organizations in especially Asia Pacific settings that share socio-economic and cultural attributes with this research context (Pakistan). An influential beginning point for this pathway is ESSL (Tuan, 2019b). ESSL can provoke corporate social responsibility within the organization by demonstrating green values through their actions and behavior. The hiring of leaders should be done with a focus on pro-environmental concerns (Li et al., 2020). Such leaders should be built at various tiers of the organizational pyramid through the recruitment process, succession planning policies, as well as development and training programs. The experience of managers of adopting environmentally specific servant behavior should be shared in the training session of leadership or *via* various communication channels within the organization (Luu, 2019). An effective rewarding system should be implemented to encourage environmentally specific servant leaders. Leaders should serve followers in pursuit of their green goals (Tuan, 2019a).

Furthermore, the organization should provide an opportunity to employees by giving them time to develop new ideas, in this way they will feel more empowered and will be motivated enough to act in accordance with the policies of the organization (Ying et al., 2020). When employees feel competent and self-determined, they feel motivated enough to identify with the organization (Tian and Robertson, 2017). Managers should understand the empowerment level of employees to create a psychologically safe environment within the workplace (Iqbal et al., 2020). For example, managers can provide employees with information regarding the environmental impact of different activities, thus can potentially signal employees’ greater support for sustainable behaviors. The result would be more empowered and satisfied workers. Managers can strengthen the conditions for empowerment by increasing opportunities for autonomous, significant, interdependent, and autonomous voluntary sustainable behaviors (Joo and Shim, 2010). For example, supporting informal group interactions and giving time to develop new ideas related to greening may empower employees to embrace voluntary green initiatives across the organization. Employees high in psychological empowerment feel more responsible to reach toward the common goal of sustainable performance (Iqbal et al., 2020). Managers should realize the competencies and capabilities of their employees and should offer them freedom in doing their tasks (Ying et al., 2020).

Green organizations are ethically esteemed and socially responsible organizations (Ababneh, 2021). The higher the identity of employees, the higher will be the motivation of employees to carry out green activities voluntarily (Das et al., 2019). Hence, employees will be more concerned regarding corporate social responsibility. The positive image of the sustainable organization in the eyes of the community makes the employees feel proud to be part of such an organization and their attachment with the organization enhances (Raza et al., 2021). Thus, the employees will be more motivated to go beyond job expectations for enhanced green functioning of the organization ultimately leading toward enhanced green organizational performance (Zafar et al., 2022a). As a result, employees of textile firms will be more motivated to perform voluntary green behavior (Nisar et al., 2021). Environmentally committed organization can have more loyal employees (Jones et al., 2014; Saleem et al., 2021). Hence, organizations more oriented toward corporate social responsibility will deem more attractive to employees and the community. Leaders of these organizations have higher responsibility on their shoulders to communicate the green vision of the organization to the employees to facilitate green organization and green behavior of employees. ESSL can play a significant role in this regard because of its altruistic nature.

### Future directions and research limitations

Despite its contribution, the present study has some limitations that should be overcome by future researchers. A limitation arises from the cross-sectional design of the present study. Although a time-lagged survey was used to draw conclusions about the casualty of the research variables, the cross-sectional research design constrains the degree to which causal inferences can be drawn (Afsar et al., 2018). A novel research path for future researchers can be to examine the framework of the present study in terms of experimental and qualitative research. Moreover, one of the limitations addresses the analysis of voluntary pro-environmental behavior in the textile industry of Pakistan. The initiatives for greening and reactions to green practices may vary across various industries like manufacturing, tourism and service industries (Tuan, 2019a). Future researchers can propagate this research stream in other cultural contexts or regions like Vietnam because this country is hierarchal in nature and culturally collectivistic (Tuan, 2019a). Respondents of such country may have higher ratings of community-oriented behaviors than those in low power distance or individualistic countries (Tuan, 2019b). Comparative analysis of the research model should be done across cultures in terms of power distance and collectivism. Furthermore, future scholars should explore what role can be played by government subsidies in enhancing organizational performance. Government subsidy is an important indicator of organizational performance (Ding et al., 2022). It should be investigated whether government subsidies can play a role in enhancing sustainability innovation for accelerating the green performance of the organization.

## Data availability statement

The raw data supporting the conclusions of this article will be made available by the authors, without undue reservation.

## Author contributions

HZ furnished the theoretical background, designed the study, contributed to the data analysis, and wrote the first draft including theoretical and practical implications. FT helped with the methodology, analysis, and discussion sections. JH helped with the improvement of the discussion section and grammar checking of the overall paper. GZ helped with editing, rewriting, and improving different sections of the paper. All authors contributed to the article and approved the submitted version.

## Conflict of interest

The authors declare that the research was conducted in the absence of any commercial or financial relationships that could be construed as a potential conflict of interest.

## Publisher’s note

All claims expressed in this article are solely those of the authors and do not necessarily represent those of their affiliated organizations, or those of the publisher, the editors and the reviewers. Any product that may be evaluated in this article, or claim that may be made by its manufacturer, is not guaranteed or endorsed by the publisher.
